# Analysis of Psychotropic Polypharmacy and Associated Factors in Antidepressant‐Treated Patients With Depressive Disorder: A Population‐Based Cohort Study Using Real World Data

**DOI:** 10.1155/da/6076326

**Published:** 2026-04-27

**Authors:** Diego Infante-Ventura, Benjamín Rodríguez-Díaz, Miguel Ángel García-Bello, Cristina Válcarcel-Nazco, Francisco Estupiñán-Romero, Francisco Javier Acosta Artiles, Beatriz González de León, Isabel Hurtado-Navarro, Tasmania del Pino-Sedeño

**Affiliations:** ^1^ Canary Islands Health Research Institute Foundation (FIISC), Tenerife, Spain; ^2^ Evaluation Unit (SESCS), Canary Islands Health Service (SCS), Tenerife, Spain, healthcareinspain.eu; ^3^ Spanish Network of Agencies for Health Technology Assessment for the National Health Service (RedETS), Madrid, Spain; ^4^ Department of Clinical Psychology, Psychobiology and Methodology, University of La Laguna, Tenerife, Spain, ull.es; ^5^ Research Network on Chronicity, Primary Care and Health Promotion (RICAPPS), Carlos III Health Institute (Instituto de Salud Carlos III), Madrid, Spain, isciii.es; ^6^ Data Science for Health Services and Policy Research Group, Aragon Health Sciences Institute (IACS), Zaragoza, Spain, iacs.aragon.es; ^7^ Department of Mental Health, General Directorate of Mental Health and Addiction, Canary Island Health Service, Las Palmas de Gran Canaria, Spain; ^8^ Department of Psychiatry, Dr. Negrín University Hospital of Gran Canaria, Las Palmas de Gran Canaria, Spain; ^9^ Management of Primary Care of Tenerife, Santa Cruz de Tenerife, Spain; ^10^ Health Services Research and Pharmacoepidemiology Unit, Foundation for the Promotion of Health and Biomedical Research of the Valencian Community (FISABIO), Valencia, Spain; ^11^ Faculty of Health Sciences, Universidad Europea de Canarias, Tenerife, Spain

## Abstract

**Introduction:**

Psychotropic polypharmacy typically refers to the prescription of more than one psychotropic medication to a patient. While polypharmacy can sometimes be clinically justified, it is widely recognized that multiple drug use and exposure can be problematic. The aim of this article is to analyze polypharmacy experienced by patients with depressive disorders, estimating its incidence during the first year after diagnosis and exploring the sociodemographic, clinical, and health‐related lifestyle factors related to psychotropic polypharmacy.

**Methods:**

A retrospective population‐based cohort study was conducted among adults (aged 18 and over) within the Canary Islands Health Service. The study analyzed routine health record data from patients diagnosed with depressive disorders who received antidepressant treatment between 2013 and 2022. Psychotropic polypharmacy refers to the concurrent use of two or more psychotropic medications by the same patient. To explore the relationship with sociodemographic, clinical, and lifestyle characteristics, multivariate logistic regression analyses were conducted.

**Results:**

The study included a total of 39,800 participants, with a polypharmacy incidence of 86.28%. Most patients (48.10%) received three or more medications (*N* = 19,145). Factors associated with polypharmacy included the 45–64 age group (OR: 1.21), previous depressive episodes (OR: 1.15), number of comorbidities (OR: 1.11), and male gender (OR: 0.83).

**Conclusion:**

The results highlight the complexity of psychotropic polypharmacy, especially in patients with previous episodes and comorbidities. While the use of multiple medications may be necessary, it is critical to periodically review treatments to ensure their safety, especially in vulnerable populations. New prescription trends suggest a shift toward more rational approaches, although further research is required to support clinical guidelines.

## 1. Introduction

Depressive disorders are severe and often recurrent disorders that deeply impact those who suffer from them. The likelihood of recurrence in patients with depression is high, with rates reaching 50% after the first episode, 70% after the second, and 90% after the third. These disorders are highly disabling and have become the leading cause of disability today [1]. Additionally, they are the largest contributor to suicide deaths. It is estimated that ~300 million people worldwide are affected by depressive disorders [2]. In Spain, depression affects 4.1% of the population, with a significantly higher incidence in women, with a ratio of 3:1 compared to men [3].

Management of depressive disorders involves diverse therapeutic options, including both pharmacological and nonpharmacological approaches. Among them, pharmacological treatment, particularly the use of antidepressants, is one of the most common and effective options for managing depression. Its effectiveness has been widely demonstrated in the general population [4]. Therefore, an appropriate prescription is crucial to ensure an effective therapeutic approach, allowing the symptoms that interfere with the patient’s quality of life to be relieved and facilitating the remission of the disease [5]. However, achieving optimal dosing is often challenging due to substantial interindividual pharmacokinetic variability and the limited availability of clinical tools, beyond pharmacogenomic testing, to guide precision prescribing [6]. Furthermore, the simultaneous prescription of multiple medications is often motivated by the need to manage a constellation of concurrent symptoms (such as anxiety or sleep disturbances), which may require different drug classes to achieve stabilization. Additionally, polypharmacy may be clinically necessary and beneficial in cases involving medical or psychiatric comorbidity, lack of response to treatment, and a complex symptom profile, as well as to maintain symptom stability and prevent relapses [7].

According to the World Health Organization (WHO), polypharmacy is defined as the simultaneous use of multiple medications. Although there is no universal definition, it is commonly recognized when a patient routinely takes five or more medications [8, 9]. In mental health, psychotropic polypharmacy involves prescribing multiple psychotropic medications concurrently to treat the same disorder, irrespective of their class or mode of action [10]. It is crucial to differentiate between appropriate and inappropriate polypharmacy. Appropriate polypharmacy is considered to exist when: (a) all medications are prescribed in order to achieve objectives agreed upon with the patient; (b) therapeutic objectives are being achieved or there is a possibility that they will be achieved in the future; (c) the regimen has been optimized to minimize the risk of adverse drug reactions; (d) the patient is motivated and able to take all medications as planned [11].

While polypharmacy can sometimes be clinically justified, it is widely acknowledged that the use of and exposure to multiple drugs are generally associated with unfavorable health outcomes that can be problematic. In the case of antidepressant treatment, the use of multiple medications increases therapeutic complexity, elevating the risk of drug–drug interactions (DDIs) [12]. DDIs occur when two or more drugs are administered simultaneously or sequentially, influencing each other’s effects on the body, and can result in unintended therapeutic effects [7]. DDIs may reduce drug efficacy and increase the likelihood of experiencing side effects, which can be severe or even fatal [13].

Polypharmacy has been associated with higher risks of side effects, adverse reactions, poor treatment adherence, and potentially dangerous drug interactions [14]. These risks are particularly concerning in cases of inappropriate polypharmacy, when prescribing one or more medications are prescribed unnecessarily or are no longer required [11]. This issue is especially significant among older adults, who, due to aging, often face a higher overall disease burden [8, 15]. However, polypharmacy in this population not only increases the risk of medication‐related complications but also contributes to an increased risk of falls, hospitalizations, and mortality [15, 16]. A recent systematic review assessing the prevalence of polypharmacy in different populations worldwide found highly worrying figures. The prevalence was 30.2%, 61.7%, and 56.9% for people in the community, hospitalized, and institutionalized patients, respectively. These findings underscore the relevance of comprehensively analyzing psychotropic polypharmacy, particularly in the current context where its prevalence is rising.

Thus, the aim of this study is to analyze a cohort of patients with a depressive disorder in the Canary Islands Health Service (Servicio Canario de la Salud, SCS) to estimate the incidence of polypharmacy and elicit patterns of psychotropic polypharmacy. Specifically, the objectives of the study are to: (1) estimate the incidence of psychotropic polypharmacy during the first year after diagnosis; (2) analyze the prevalence, distribution, and temporal trends of psychotropic medication prescriptions by polypharmacy status; (3) explore the sociodemographic, clinical, and lifestyle factors associated with psychotropic polypharmacy. Real‐world data were analyzed to achieve the objectives of the present study. By evaluating these data, the authors seek to estimate its incidence during the first year, identify its typology, and provide insights into its implications for clinical practice and mental health policy.

## 2. Methods

This research is part of the project titled “*Utilización de datos sanitarios de vida real para el análisis de la adherencia a la terapia farmacológica de pacientes con trastorno depresivo (Using real-life health data to analyze adherence to drug therapy in patients with depressive disorder)*,” funded by the Canary Islands Health Research Institute Foundation (PIFIISC20_05). The project aims to analyze therapeutic adherence, polypharmacy, care trajectories, and treatment costs in patients with depressive disorders. Detailed methods and population characteristics have been described in a previous publication [17]. This study was reported in accordance with the STROBE statement (Table S1 for the completed checklist of items recommended for cohort study reports).

### 2.1. Design and Setting

A retrospective cohort study at the population level was conducted using routinely collected administrative and clinical records from all primary and specialized care healthcare centers of the SCS. Given the integrated nature of the Spanish public health system, patients often receive shared care from both general practitioners and specialists. Therefore, data from all levels were aggregated to analyze each patient’s total medication burden, regardless of the prescribing provider. The study covered the period from 2013 to 2022.

### 2.2. Participants

The inclusion criteria were: (1) age 18 years or older; (2) a diagnosis of depressive disorder according to ICD‐10 codes F32–F33: depressive episodes and recurrent depressive disorder, or F34.1: dysthymia (see Table S2 for ICD‐9 equivalents); (3) initiation of treatment with an antidepressant (Anatomical Therapeutic Chemical [ATC] code: N06A); (4) registration in the electronic health records (EHRs) of the SCS between 2013 and 2020.

Patients treated exclusively with nonpharmacological interventions or psychotropic medications without concomitant antidepressant use were not included in the analysis.

Patients were excluded if they had a previous diagnosis of manic episode or bipolar disorder, or schizophrenia, schizotypal or delusional disorders (ICD‐10: F30–F31 and F20–F29, respectively; corresponding ICD‐9 codes are provided in Tables S3 and S4).

### 2.3. Variables

#### 2.3.1. Primary Outcome

Psychotropic polypharmacy, which was defined as the concurrent use of two or more psychotropic medications by the same patient [10, 18], was selected as the primary outcome measure for this study.

The classification of psychotropic polypharmacy proposed by the National Association of State Mental Health Program Directors (2001) was partially applied, which encompasses five types of polypharmacy: same‐class polypharmacy, multiclass polypharmacy, adjuvant, augmentation, and total polypharmacy [19].

• Same‐class (within‐class) polypharmacy refers to the use of more than one medication from the same pharmacological class.
• Multiclass (between‐class) polypharmacy refers to the use of full therapeutic doses of more than one medication from several pharmacological classes for the same symptom group.
• Total polypharmacy refers to the total number of medications used in a patient or the total medication load.
• Adjuvant and augmentation polypharmacy were not assessed due to the retrospective nature of the data.

Prescription data for antidepressant treatment (ATC code: N06A) and other psychoactive medications—including antiepileptics (N03A), antipsychotics (N05A), anxiolytics (N05B), and hypnotics/sedatives (N05C)—were collected for a 12‐month period following patients´ inclusion in the cohort.

#### 2.3.2. Characterization Measures

Characterization data for each patient were retrieved from information available at the time of their initial antidepressant prescription following cohort entry. Sociodemographic variables included age (derived from date of birth) and sex, alongside lifestyle‐related factors such as smoking status, alcohol intake, substance abuse, physical activity, and dietary patterns. Clinical variables included diagnostic criteria based on ICD‐10 codes for depressive disorders (F32–F33) or dysthymia (F34.1), number of previous depressive episodes, and the presence of medical and psychotropic comorbidities (Table 1) as registered in their EHR.

**Table 1:** Characteristics of participants and prevalence of psychotropic polypharmacy.

Characteristics	Total	Polypharmacy	No polypharmacy	*p*‐Value
Patients, *N*	39,800	34,338 (86.28)	5,462 (13.72)	—
Prescriptions, *N*	693,686	—	—	—
Age, mean (SD)	56.28 (15.84)	56.29 (15.55)	56.18 (17.57)	0.914
Age, *N* (%)	—	—	—	—
18–44 yrs.	9603 (24.13)	8098 (23.58)	1505 (27.55)	<0.001
45–64 yrs.	17,925 (45.04)	15,790 (45.98)	2135 (39.09)	<0.001
65–74 yrs.	6048 (15.20)	5266 (15.34)	782 (14.32)	0.051
>75 yrs	6224 (15.64)	5184 (15.10)	1040 (19.04)	<0.001
Gender, *N* (%)	—	—	—	<0.001
Male	29,143 (73.22)	25,342 (73.80)	3801 (69.59)	<0.001
Female	10,657 (26.78)	8996 (26.20)	1661 (30.41)	<0.001
Number of drugs, median (IQR)	2.0 (2.0–3.0)	3.0 (2.0–3.0)	1.0 (1.0–1.0)	<0.001
Number of drugs, *N* (%)	—	—	—	—
1 drug	5462 (13.72)	—	5462 (100.00)	<0.001
2 drugs	15,193 (38.17)	15,193 (44.25)	—	<0.001
≥3 drugs	19,145 (48.10)	19,145 (55.75)	—	<0.001
Prescribed days	53.60 (29.78)	66.08 (40.19)	51.62 (27.24)	<0.001
Diagnosis, *N* (%)	—	—	—	<0.001
Single episode	32,870 (82.59)	28,352 (82.57)	4518 (82.72)	0.787
Recurrent	212 (0.53)	204 (0.59)	8 (0.15)	<0.001
Dysthymic disorder	6718 (16.88)	5782 (16.84)	936 (17.14)	0.585
Lifestyle, *N* (%)	36,155	31,209	4,946	—
Inadequate nutrition	16,942 (46.86)	14,708 (47.13)	2234 (45.17)	0.010
Inactive physical activity	16,746 (46.32)	14,510 (46.49)	2236 (45.21)	0.092
Tobacco user	4107 (11.36)	3618 (11.59)	489 (9.89)	<0.001
Drug user	3018 (8.35)	2618 (8.39)	400 (8.09)	0.477
Alcohol user	2956 (8.18)	2530 (8.11)	426 (8.61)	0.227
Prior depressive episodes	0 (0–1)	0 (0–1)	0 (0–1)	<0.001
Medical comorbidities, N (%)	33,242	28,836	4,406	—
Dyslipidaemia	22,373 (67.30)	19,532 (67.73)	2841 (64.48)	<0.001
Hypertension	20,522 (61.74)	17,901 (62.08)	2621 (59.49)	<0.001
Obesity	10,823 (32.56)	9376 (32.51)	1447 (32.84)	0.666
Diabetes mellitus	9717 (29.23)	8409 (29.16)	1308 (29.69)	0.475
Migraine	3547 (10.67)	3092 (10.72)	455 (10.33)	0.428
Fibromyalgia	3099 (9.32)	2811 (9.75)	288 (6.54)	<0.001
Asthma	2364 (7.11)	2089 (7.24)	275 (6.24)	0.016
Hearing loss	2358 (7.09)	2018 (7.00)	340 (7.72)	0.084
Fatigue	1245 (3.75)	1084 (3.76)	161 (3.65)	0.732
Hypertensive heart disease	1102 (3.32)	973 (3.37)	129 (2.93)	0.123
Psychiatric comorbidities, N (%)	39,788	34,326	5,462	—
Anxiety disorders	7387 (18.57)	6679 (19.46)	708 (12.96)	<0.001
Other mental disorders^a^	6333 (15.92)	5828 (16.98)	505 (9.25)	<0.001
Sleep disorders	4094 (10.29)	3784 (11.02)	310 (5.68)	<0.001
Nicotine dependence	2782 (6.99)	2450 (7.14)	332 (6.08)	0.004
Impulse‐control disorders	1462 (3.67)	1366 (3.98)	96 (1.76)	<0.001
Reaction to severe stress	1258 (3.16)	1135 (3.31)	123 (2.25)	<0.001

*Note*: *N*: number of patients.

Abbreviations: IQR, interquartile range; SD, standard deviation; yrs., years.

^a^“Other Mental Disorders” is defined as any comorbid diagnosis of a mental disorder (ICD‐10 F codes) that is not individually listed in this table or in the exclusion criteria, such as personality disorders (F60–F69), eating disorders (F50), and somatoform disorders (F45).

### 2.4. Data Sources

Data for this study were sourced from administrative and EHRs from Primary Care and Specialized Care (DRAGOAP and DRAGOAE, respectively), as well as the Canary Islands Registry of Psychiatric Cases (RECAP) and the continuous electronic prescription system.

### 2.5. Analysis

**Figure 1:**
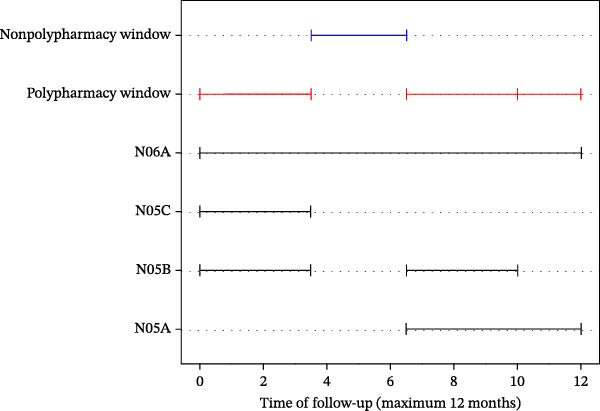
Drug use and polypharmacy windows.

The different definitions of polypharmacy were derived from the patient prescriptions database. During the 12 months following each patient’s inclusion in the cohort, the time windows in which patients were prescribed a polypharmacy regimen were identified, as well as the periods (or time windows) in which they did not undergo polypharmacy (Figure 1). A patient was classified as experiencing polypharmacy if they had at least one time window with polypharmacy within this period. Conversely, a patient was considered nonpolypharmacy if, throughout the entire 12‐month period, they were prescribed only one medication at a time. Analyses were subsequently conducted at both the time‐window and patient levels.

Descriptive analyses were performed for patients stratified by polypharmacy status. Frequencies and percentages were used to present categorical variables, while means and standard deviations (or medians and interquartile ranges) were used for numerical variables. For group comparisons, normally distributed continuous variables were analyzed using the *t*‐test, whereas the Wilcoxon rank‐sum test was applied to nonnormally distributed variables. Categorical variables were evaluated with the χ^2^ test, or Fisher’s exact test for variables with expected frequencies below 5%.

In order to evaluate patterns of antidepressant use, frequencies, and percentages (with 95% confidence intervals (95% CI) were calculated separately for both prescriptions and patients, by polypharmacy status (including types of polypharmacy) and drug group.

Logistic regression models (constructed using the “backward” method) were used to determine which factors most influenced polypharmacy. Covariates included age (categorical), sex, health‐related lifestyle behaviors (tobacco, alcohol, and drugs), number of comorbidities (continuous), and previous episodes (continuous).

Temporal trends (2014–2021) in the patterns of psychoactive medication prescription for patients with depressive disorders were analyzed. Annually, the percentage of patients prescribed each drug group was analyzed, along with trends in polypharmacy and specific drug combinations. To assess these trends, proportions overtime were compared using the χ^2^ test. The results were plotted to show the annual distribution of prescriptions for the total sample and stratified by adherence status. Statistical significance was set at *p*  < 0.05, and all analyses were conducted in R 4.3.3 [20].

### 2.6. Ethics

This study was based on retrospective, pseudonymized data from the EHRs that were nonidentifiable and nontraceable, provided by the SCS. The protocol was approved by the Research Ethics Committee of the University Hospital of the Canary Islands (CHUNSC_2020_56) on June 26, 2020. Informed consent was not required, as the study fulfilled the exemption criteria outlined in Spanish legislation—specifically, Royal Decree 957/2020 of November 3—regarding observational studies involving medicinal products for human use. Ethical procedures and legal compliance have been thoroughly detailed in a previous publication [17].

## 3. Results

Of the 39,800 participants included in the study, 73.22% were women. The mean age of the cohort was 56.28 ± 15.84 years. The study analyzed 693,686 prescriptions in total. The overall prevalence of polypharmacy among participants was 86.28%. Sociodemographic, clinical, and lifestyle characteristics are summarized in Table 1, stratified by polypharmacy status. Although there was no difference in mean age between the two groups (*p* = 0.91), the age distribution differed significantly (*p*  < 0.001). Specifically, the relationship between age and medication burden followed a nonlinear pattern, characterized by a peak in the prevalence of polypharmacy in the 45–64 age group (45%). After this peak, polypharmacy decreased in the older age group (>75 years), where monotherapy became more common (19.04%) compared with middle‐aged adults (Table 1).

Regarding medication use, the largest group consisted of patients who received three or more drugs, accounting for 48.10% of the total (*N* = 19,145). This was followed by the group that received two drugs, comprising 38.17% of the total (*N* = 15,193), and finally, the group that received only one drug, representing 13.72% of the total (*N* = 5462).

In the analysis of antidepressant use distribution among 693,686 prescriptions and 39,800 patients, a clear preference was observed for selective serotonin reuptake inhibitors (SSRIs, ATC code N06AB) (Table S5). SSRIs accounted for 45% of the prescriptions and were used by ~69% of the patients. Among these, sertraline was the most commonly prescribed drug, accounting for 14% of the prescriptions and used by 22% of the patients, followed by escitalopram at 10% per prescriptions and 18% per patients, and paroxetine at 9% and 16%, respectively.

Second, antidepressants classified under ATC code N06AX were the most prescribed group, accounting for 50% of prescriptions and used by 55% of the patients. The most frequently prescribed medications in this group were trazodone (11% per prescriptions; 18% per patients), mirtazapine (10%; 15%), and duloxetine (8%; 13%).

Last, nonselective monoamine reuptake inhibitors (N06AA) were the least used group, accounting for 5% of prescriptions and used by 9% of the population. Amitriptyline was the predominant drug in this group, with 4% per prescriptions and a 7% usage rate per patients. Table 2 presents the distribution of antidepressant use.

**Table 2:** Number of patients by psychotropic polypharmacy.

ATC code	Group description	Polypharmacy *N* = 34 338 (%; CI 95%)	No polypharmacy *N* = 5462 (%; CI 95%)
N06A	Antidepressants	34,338 (100)	5,462 (100)
N05B	Anxiolytics	28,288 (82.38; 81.97–82.78)	—
N05C	Hypnotics and sedatives	12,808 (37.30; 36.79–37.81)	—
N05A	Antipsychotics	4780 (13.92; 13.56–14.29)	—
N03A	Antiepileptics	1189 (3.46; 3.27–3.66)	—

*Note:* represents the total number of patients who consumed that medication at any point during the year. *N*: number of patients.

Abbreviation: CI 95%: 95% confidence interval.

Given that the initiation of an antidepressant (N06A) was a mandatory inclusion criterion for the cohort, all participants across both groups utilized medications from this class (100% prevalence). Consequently, the no polypharmacy group comprises patients managed exclusively with antidepressant monotherapy, whereas the Polypharmacy group includes those who used an antidepressant concurrently with at least one other psychotropic medication. (Supporting Information Table S6 for a complete list of medications included). In patients with total psychotropic polypharmacy, the most frequently used drug group was anxiolytics (N05B), accounting for 82%, followed by hypnotics and sedatives (N05C) at 37%, antipsychotics (N05A) at 14%, and antiepileptics (N03A) at 4% (Table 2).

Similarly, among prescriptions involving total psychotropic polypharmacy, anxiolytics (N05B) remained the most frequently used group at 32%, followed by hypnotics and sedatives (N05C) at 12%, antipsychotics (N05A) at 4%, and antiepileptics (N03A) at 1% (Table 3).

**Table 3:** Number of prescriptions by psychotropic polypharmacy.

ATC code	Group description	Polypharmacy	No polypharmacy
		*N* = 667 271 (%; CI 95%)	*N* = 26 415 (%; CI 95%)
N06A	Antidepressants	340,001 (50.95; 50.83–51.07)	26,415 (100.00)
N05B	Anxiolytics	212,693 (31.88; 31.76–31.99)	—
N05C	Hypnotics and sedatives	82,623 (12.38; 12.30–12.46)	—
N05A	Antipsychotics	23,530 (3.53; 3.48–3.57)	—
N03A	Antiepileptics	8424 (1.26; 1.24–1.29)	—

*Note: N*: number of patients.

Abbreviation: CI 95%: 95% confidence interval.

The analysis of the different therapeutic regimens showed that monotherapy was used in 19.07% of cases, while some type of psychotropic polypharmacy was present in 80.93% of the population, referring to the total time each patient was taking medication. Within the different psychotropic polypharmacy regimens, polypharmacy within‐class (same‐class polypharmacy) occurred in 6.5% of cases, between‐class polypharmacy (multiclass polypharmacy) was the most frequent with 46.5%, and the combination of both strategies was present 28% of the time (Table 4).

**Table 4:** Monotherapy and psychotropic polypharmacy classification by time windows (by gender and age).

Variable	Monotherapy *N* (%)	Within‐class polypharmacy *N* (%)	Between‐class polypharmacy *N* (%)	Both (within and between) *N* (%)	Total polypharmacy *N* (%)
Per time window (*N* = 279,264)	53,263 (19.07)	18,051 (6.46)	129,839 (46.49)	78,111 (27.97)	226,001 (80.93)

Gender					

Female (*N* = 29,143)	22.27	5.33	48.98	23.42	77.73
Male (*N* = 10,657)	25.02	6.72	46.17	22.08	74.98

Age (yrs.)					

18–44 (*N* = 9603)	27.58	5.25	47.47	19.71	72.42
45–64 (*N* = 17,925)	20.51	5.52	48.23	25.74	79.49
65–74 (*N* = 6048)	20.43	5.61	49.68	24.27	79.57
>75 (*N* = 6224)	25.64	7.04	47.98	19.34	74.36

*Note: N*, number of patients.

Abbreviations: SD, standard deviation; yrs., years.

In terms of gender distribution, women had a higher percentage of time in total psychotropic polypharmacy compared to men. Specifically, women used monotherapy 22% of the time. Between‐class polypharmacy was the regimen that covered the largest percentage of time at 49%, followed by the combination of within‐ and between‐class polypharmacy at 23% and within‐class polypharmacy at 5% of the time. Men, on the other hand, used monotherapy 25% of the time, and between‐class polypharmacy was also the most common at 46%, followed by the combination of strategies at 22% and within‐class polypharmacy at 7%.

In the age analysis, the time in total psychotropic polypharmacy increased with age up to 75 years. In the 18–44 age group, monotherapy accounted for 28% of the time, while total polypharmacy accounted for 72%. In this group, between‐class polypharmacy was the most frequent (48%), followed by the combination of strategies (20%) and within‐class polypharmacy (5%). In the 45–64 and 65–74 age groups, total polypharmacy was dominant, with values around 79%. Between‐class polypharmacy predominated, followed by the combination of strategies (within‐ and between‐class polypharmacy) and polypharmacy within‐class. In those over 75 years of age, monotherapy accounted for 26% of the time, which was higher than in the 45–75 age group and similar to that in the younger age group. Although polypharmacy within the same class presented higher percentages of time than the rest of the groups, the combination of strategies had the lowest percentage of time. Table 4 presents the classification of time windows and patients based on monotherapy and different types of psychotropic polypharmacy, stratified by gender and age groups.

### 3.1. Analysis of Polypharmacy Predictors

In the adjusted analysis using multivariable logistic regression of the factors associated with polypharmacy (Table 5), age showed a significant relationship, with an increase of polypharmacy in the 45–64 age group, while individuals aged 75 and over showed a decrease. Tobacco and alcohol use was also positively associated with polypharmacy. Furthermore, the risk of polypharmacy increased with each additional previous episode and with each additional comorbidity. Male sex was associated with a lower risk of polypharmacy compared to female sex. Nutrition, alcohol consumption, and the 65–75 age group were not significantly associated with polypharmacy.

**Table 5:** Factors associated with polypharmacy.

Characteristic	OR	95% CI	*p*‐Value
Sociodemographic data	—	—	—
Age	—	—	<0.001
18−44 yrs.	—	—	—
45−64 yrs.	1.21	1.12, 1.30	<0.001
65−74 yrs.	1.00	0.90, 1.11	0.989
>75 yrs.	0.73	0.67, 0.81	<0.001
Sex	—	—	<0.001
Woman	—	—	—
Man	0.83	0.78, 0.88	<0.001

Clinical data			

Previous depressive episodes	1.15	1.09, 1.21	<0.001
Number of comorbidities	1.11	1.10, 1.12	<0.001

Health‐related lifestyle behaviors			

Nutrition	—	—	0.111
Adequate	—	—	—
Inadequate	0.95	0.90, 1.01	0.111
Tobacco	—	—	0.02
No tobacco	—	—	—
Tobacco	1.14	1.02, 1.27	0.02
Alcohol	—	—	0.07
Abstemious	—	—	—
Drinker	0.90	0.80, 1.01	0.06

Model pseudo‐*R* ^2^ = 0.026			

Abbreviations: 95% CI, 95% confidence interval; OR, odds ratio; yrs., years.

### 3.2. Analysis of Temporal Trends

**Figure 2:**
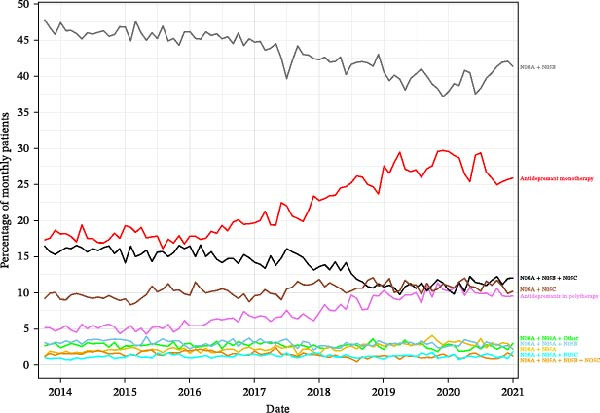
Temporal trend by psychoactive drug group.

Figure 2 shows the percentage of patients overtime (month‐over‐month) receiving different drug combinations. The most notable trend is the decreasing use of the combination “Antidepressants + Anxiolytics,” which starts at about 48% of patients in 2014 and declines to about 42% in 2021. In contrast, the use of antidepressant monotherapy shows a significant increase, rising from about 15% in 2014 to about 28% in 2021. The combination of antidepressants also shows a notable increase in use over this period. The remaining combinations appear to be used in a much smaller and relatively stable percentage of patients over the years. Overall, the data suggest a shift in treatment practices, moving toward monotherapy or polypharmacy with antidepressants (N06A) and away from other combined treatment options.

## 4. Discussion

In this population‐based study, we analyzed psychotropic polypharmacy among patients with depressive disorders treated in the SCS. The main objectives were to determine the prevalence and temporal trends and to identify associated sociodemographic, clinical, and lifestyle factors.

Direct comparison of polypharmacy incidence across studies is often limited by the specific criteria used to define it. Two main factors influence the reported rates: the numerical threshold for polypharmacy (e.g., two or more versus five or more) and the scope of drugs considered (e.g., psychiatric‐specific medications versus any concomitant medications). These methodological variations inherently affect the comparability of findings and should be taken into account when interpreting incidence data.

Among the 39,800 antidepressant‐treated patients with depressive disorders included in the study, the estimated overall incidence of psychotropic polypharmacy was ~86%, an alarmingly higher figure than previously reported. A previous study reported the prevalence of psychotropic polypharmacy, defined as ≥2 psychotropic medications, at 58% during the 2014–2015 period [21]. On the other hand, two other pharmacovigilance studies in patients with depressive disorders estimated that overall polypharmacy, defined as ≥5 concurrent medications, occurred in 23% and 31% of cases [22, 23]. While a systematic review, not limited to psychotropic polypharmacy using a threshold of ≥5 medications, reported a lower prevalence of 37% [24].

Approximately 38% of patients were prescribed two psychotropic medications, while 48% were on regimens involving three drugs. While this pattern could potentially reflect an attempt to optimize treatment efficacy in response to clinical challenges (such as multiple comorbidities, previous episodes, or potentially treatment‐resistant depression), it is important to note that the available data do not allow for a definitive conclusion on the clinical rationale behind these prescribing choices. Other factors, including the management of specific residual symptoms or variations in clinical practice, may also contribute to the complexity of the regimens, requiring a more cautious interpretation of the reported findings. Among patients receiving psychotropic polypharmacy, anxiolytics (82%), and hypnotics and sedatives (37%) were the most commonly prescribed, followed by antipsychotics (14%) and antiepileptics (4%). These prescribing trends observed in the present study suggest a predominant use of medications targeting anxiety and sleep disorders, probably reflecting the high burden of psychiatric symptoms in this population [25, 26]. Given these prescribing trends, it is crucial to evaluate whether the combinations of psychotropic medications are clinically justified. A previous study by Rhee and Rosenheck [21] conducted a detailed analysis of psychotropic polypharmacy, evaluating the clinical justification for between‐drug combinations. Their study found that the combination of antidepressants and antipsychotics in the treatment of depression was clinically justified in 100% of the patients evaluated, supporting the therapeutic validity and effectiveness of the combined strategy in this context. However, when it came to combining antidepressants with hypnotics and sedatives from different pharmacological classes, only 30% of the patients had appropriate clinical indications for such treatment. Moreover, only 11% of the patients received a justified combination of antidepressants and mood stabilizers, highlighting the need for a careful evaluation of the risk–benefit balance to optimize health outcomes for the patients. However, the present study found that this prescription rate exceeds the proportion of patients diagnosed with comorbid anxiety (18%) and sleep disorders (10%) in the study population. This discrepancy suggests that not all instances of polypharmacy can be justified as individualized treatments for comorbid conditions, raising concerns about the appropriateness of some prescriptions. Meanwhile, the notable presence of antipsychotics and antiepileptics in treatment regimens may indicate a subset of patients with more complex management needs, requiring broader pharmacological strategies—although the use of antiepileptics in this context is less well‐supported by existing evidence [27, 28].

It is worth noting that the use of two or more psychotropic medications at the same time may not be the most appropriate approach, particularly for vulnerable populations such as adults over 65 years old [29]. However, it is also relevant that the findings here indicate a decrease in such polypharmacy specifically for individuals aged 75 and over. Despite these concerns, the present study found that more than 85% (*N* = 10,450) of patients within this age group were prescribed a psychotropic polypharmacy regimen, defined as receiving at least two psychotropic prescriptions. The aforementioned decrease in polypharmacy among people aged 75 and over may suggest that this approach may already be taking place, impacting this specific subgroup, a practice that should be strengthened and expanded. These findings underscore the need for a careful and ongoing review of treatment regimens to ensure they are both effective and safe, balancing symptom control with the potential risks associated with polypharmacy.

Beyond evaluating the justification of psychotropic drug combinations, it is equally important to consider how polypharmacy influences treatment adherence. A previous study has found that the prevalence of polypharmacy is higher in women [23], a finding that aligns with both the present study and existing literature [21, 30]. While some of these differences may be justified by variations in disease prevalence, biological factors or treatment needs, others are more challenging to explain on medical grounds and may suggest gender inequalities in prescribing practices in this context [31–33]. An in‐depth interpretation of these findings requires examining factors associated with therapeutic adherence, as they may help explain variations in patients’ likelihood of following prescribed treatment regimens.

It is interesting to consider the clinical implications of individual metabolic variability, particularly in relation to treatment adherence. There is growing evidence that much of the interindividual response to psychotropic drugs lies in genetically determined enzyme activity. The metabolism of antidepressants is closely associated with the activity of the CYP2D6 and CYP2C19 enzymes, which significantly influence the variability in plasma levels and the tolerability of these medications [34–36]. Specifically, patients with a poor metabolizer phenotype for CYP2D6 are at a higher risk of experiencing adverse effects [34]. Additionally, among young adults, being a poor metabolizer of either CYP2C19 or CYP2D6 is linked to an increased rate of treatment changes, psychiatric emergencies, and instances of self‐harm while using sertraline, fluoxetine, or (es)citalopram [37]. Similar, though more modest, results have been found in late‐life depression [38]. Incorporating genetic testing to identify patients’ enzyme activity profiles before prescribing could significantly improve treatment adherence and outcomes by providing tailored medication plans. Implementing such practices could lead to more personalized and effective healthcare strategies [35].

A goal of contemporary biomedical research is to understand the mechanisms underlying sexual dimorphism. To achieve this, researchers have attempted to integrate findings from both clinical studies in humans and experimental research in animal models are integrated in order to explain the physiological and pharmacological bases of observed sex disparities [39–42]. In this context, sex‐related differences in pharmacokinetics may emerge as a contributing factor. Emerging evidence suggest that women may metabolize antidepressants differently from men, although these findings remain inconclusive. Key factors involved include lower hepatic blood flow, a higher percentage of body fat, and differences in the activity of cytochrome P450 enzymes (particularly CYP3A4 and CYP1A2), which could lead to higher systemic drug concentrations in females [39, 41]. However, metabolic differences are not limited to these constitutive factors. The influence of sex steroid hormones (particularly estrogens) adds an additional layer of complexity to sexual dimorphism in antidepressant response. Estrogens can modulate both the expression and activity of various cytochrome P450 isoenzymes, as well as drug transport proteins such as P‐glycoprotein [40, 41]. This modulation may either exacerbate or mitigate baseline differences in the metabolism and clearance of these drugs. In addition, estrogens exert relevant neuromodulatory effects by interacting with neurotransmission systems such as the serotonergic, noradrenergic, and dopaminergic pathways, which are the main therapeutic targets of psychotropic drugs used to treat depression [39]. Ultimately, the pharmacokinetic and neuromodulatory differences in antidepressant response between women and men create a complex and dynamic scenario. Understanding these mechanisms is essential for progressing toward personalized pharmacotherapy that considers biological sex as a key variable in the efficacy and safety of treatment.

The study found that the probability of receiving polypharmacy was higher in patients under 65 years of age, and these findings align with a study by Rhee and Rosenhek [21], which analyzed the characteristics of patients visiting a psychiatrist revealing that polypharmacy was most common among adults under 65 years of age, accounting for 86.6% of cases. Although polypharmacy is often associated with older adults, given the high comorbidity with other somatic diseases, it remains a significant concern among younger psychiatric patients as well, underscoring the complexity of prescribing patterns across different age groups. However, apart from age, other clinical factors may also play a critical role in determining the likelihood of polypharmacy.

In particular, this study found that the risk of polypharmacy increased with each additional previous episode and comorbidity, reinforcing the well‐established association between comorbidities and polypharmacy observed in previous studies [23, 28]. In terms of medical comorbidities, conditions such as obesity, dyslipidaemia, and metabolic syndrome not only increase the burden of disease but are also linked to a higher risk of psychotropic polypharmacy [43, 44]. As expected, psychiatric comorbidities such as dementia [45], Alzheimer’s disease [46], schizophrenia [44, 46], and personality disorders [47] are consistently associated with an increase in psychotropic polypharmacy. Notably, the association between polypharmacy and comorbidities appears to persist regardless of whether medical or psychiatric conditions are considered.

Furthermore, certain lifestyle factors showed associations with polypharmacy. These findings align with prior research suggesting that some lifestyle‐related factors, such as physical activity and tobacco use, may be associated with polypharmacy patterns, in conjunction with other determinants not studied here, such as being overweight and socioeconomic status [23]. In addition, other studies have found consistent associations between substance use/abuse and polypharmacy [48, 49]. While the factors identified in our multivariate model acted as significant predictors of polypharmacy, the proportion of unexplained variance suggests the possible influence of other unmeasured factors. This high residual variability is expected, especially in large samples like ours, and can be attributed to the inherent heterogeneity of the studied population [50, 51]. In clinical practice, the variables used by healthcare prescribers do not always allow for the systematic or structured capture in EHRs of aspects such as symptom severity or treatment resistance, among others.

Finally, regarding temporal trends in prescriptions, the findings in this study suggest a potential change in traditional prescribing patterns. The increase in prescriptions of antidepressants and antipsychotics might imply a shift toward more versatile treatment approaches and alternative therapeutic strategies. Notably, the decrease in the use of anxiolytics could be postulated as an example, being eliminated or replaced by certain dual‐action antidepressants (e.g., mirtazapine) or by low doses of antipsychotics (e.g., quetiapine) [52, 53. In accordance with previous scientific literature that proposes the reevaluation of the role of anxiolytics in long‐term treatments [54, 55]. In the Spanish context, the corresponding health authorities have initiated a deprescription campaign for anxiolytics, encouraging prescribers to discontinue their prescriptions, with the aim of reversing the growing trend [56]. Future studies are needed to assess whether the transition to antidepressant monotherapy or polytherapy, described earlier in our findings, actually translates into better tolerability and improved clinical response rates in the population.

### 4.1. Strengths and Limitations of the Study

A key strength of this research lies in the analysis of the complete population of a region in Spain, as it covers all patients with depressive disorders who initiated treatment with an antidepressant in the Canary Islands Health System. Unlike previous studies, another notable advantage is the inclusion of data from all levels of public healthcare, encompassing primary care, specialized care, hospitalizations, and emergency departments. This comprehensive approach allows for a more holistic understanding of the management of depressive disorders within the health system.

An alternative research design was used to gather more evidence on outcomes associated with psychotropic polypharmacy, which is difficult to assess in RCTs, where comparing psychotropic polypharmacy regimens can be a time‐consuming and complex process. Moreover, the use of real‐world data offers valuable insights into personalized medicine as a key component of effective psychiatric care, recognizing the variability in patient responses to multiple medications. This approach aligns with the principles of evidence‐based medicine, which emphasizes integrating clinical expertise with high‐quality external evidence [57].

However, several limitations should be considered. First, the nature of the retrospective design and the use of routine records impose certain limitations of their own. However, in an attempt to compensate for this, a systematic and rigorous effort has been made to integrate data from various sources. Second, there is the possibility of data bias, derived from the uniformity in the collection of information by health care providers. While large databases offer valuable insights, the significant heterogeneity in how information is recorded across professionals may reduce the accuracy of real‐world data representation. Another limitation of the study, due to the inherent characteristics of the available data, was the inability to analyze polypharmacy in treatment‐resistant or relapsed patients, as well as adjuvant and augmentation polypharmacy. It should be noted, however, that the study’s objective was not medication adherence, as assessed in a previous article [17], nor was it to evaluate treatment suitability, as this will be assessed in a subsequent article. Furthermore, while our analysis required temporal overlap to define polypharmacy, distinguishing between intentional coprescription and cross‐tapering periods during medication switches remains challenging using administrative data. Moreover, prescription records indicate medication possession rather than confirmed daily consumption; therefore, we cannot definitively differentiate between continuous concurrent use and “as needed” administration, particularly for anxiolytics. Additionally, because inclusion required the initiation of antidepressant treatment, our estimates of polypharmacy incidence apply specifically to the pharmacologically treated population and not to all individuals diagnosed with depressive disorders (e.g., those managed solely with psychotherapy). Finally, and related to polypharmacy, the study only focused on psychiatric medication, so it did not include other medications, both prescribed and over‐the‐counter, which could have led to an underestimation of the simultaneous use of drugs with antidepressants. This limits analysis of interactions between psychotropic and somatic drugs (e.g., for diabetes or hypertension), which is particularly relevant for our cohort, given their mean age (56 years) and comorbidities, both of which increase the risk of polypharmacy. Since DDIs represent a major safety concern, they should be specifically addressed in future research.

In order to address these challenges, future studies should focus on standardizing data collection methods through national and international registries, ensuring harmonized healthcare data for more reliable findings. A massive data collection could serve as a crucial resource for enhancing responsiveness and evidence‐based decision‐making. Furthermore, additional research is needed to explore the reasons behind these prescribing trends and to examine the broader factors associated with psychotropic polypharmacy. Comparative analyses between polymedicated and nonpolymedicated patient populations would also provide a more precise understanding of this phenomenon and its clinical implications. Finally, a key priority for future research is the development of a standardized and operational definition of polypharmacy. Rather than endorsing a single universal threshold, such a definition could support the use of context‐specific numerical cut‐offs, adapted to different clinical scenarios, thereby improving the comparability and applicability of findings across diverse patient populations and healthcare settings.

## 5. Conclusion

The results presented highlight the complexity of psychotropic polypharmacy, especially in patients with previous episodes and multiple comorbidities. While the use of concurrent medications may be necessary to improve treatment efficacy, it is crucial to periodically review therapeutic regimens to ensure their safety, particularly in vulnerable populations. The variability in the definition of polypharmacy and in data collection highlights the need for standardized approaches in research. Furthermore, emerging trends in prescribing, such as the use of antidepressants and antipsychotics instead of anxiolytics, suggest a possible transition toward more rational approaches to treatment. However, further research is essential to delve deeper into these trends and provide a solid basis for improving clinical prescribing guidelines.

## Author Contributions

**Diego Infante-Ventura**: writing – original draft, investigation, formal analysis. **Benjamín Rodríguez-Díaz**: writing – original draft, foftware, formal analysis, data curation. **Miguel Ángel García-Bello**: software, data curation, writing – review and editing. **Cristina Válcarcel-Nazco and Francisco Estupiñán-Romero**: conceptualization, methodology, writing – review and editing, supervision. **Francisco Javier Acosta Artiles, Beatriz González de León, and Isabel Hurtado-Navarro**: conceptualization, methodology, writing – review and editing. **Tasmania del Pino-Sedeño**: conceptualization, methodology, investigation, writing – review and editing, supervision, project administration, funding acquisition.

## Funding

The authors declare financial support was received for the research, authorship, and/or publication of this article. This work has been carried out within the framework of a project financed by the Canary Islands Health Research Institute Foundation (FIISC) in the Call for Research, Development and Innovation Projects aimed at satisfying the health needs of the population of the Canary Islands and improving the sustainability and solvency of the Canary Health Service (SCS) (Grant PIFIISC20_05).

## Disclosure

All authors read and approved the final version of the manuscript. All AI‐generated content was carefully reviewed and validated by the authors prior to submission.

## Ethics Statement

The study protocol was approved by the Research Ethics Committee of Hospital Universitario de Canarias ([CHUNSC_2020_56] decision June 26, 2020).

## Consent

According to this legislation and ethical standards, obtaining informed consent from patients is not obligatory for observational studies containing no directly identifiable patient data (17 of Royal Decree 957/2020, of November 3, which regulates observational studies with medicines for human use).

## Conflicts of Interest

The authors declare no conflicts of interest.

## Supporting Information

Additional supporting information can be found online in the Supporting Information section.

## Supporting information


**Supporting Information** The supporting information includes the STROBE Statement (Table S1) and correspondence tables between ICD‐10 and ICD‐9 diagnostic codes for depressive disorders (Table S2), manic episode and bipolar disorder (Table S3), and schizophrenia, schizotypal disorder, and delusional disorders (Table S4). Furthermore, Supporting Table S5 provides a distribution of antidepressant use by prescription and by patient, with an analysis according to psychotropic polypharmacy/no‐polypharmacy.

## Data Availability

The datasets analyzed during the current study are not publicly available due to institutional data protection policies but are available from the corresponding author upon reasonable request.

## References

[1] NICE , *Depression in Adults: Treatment and Management*. National Institute for Health and Care Excellence: Guidelines, National Institute for Health and Care Excellence (NICE), 2022, http://www.ncbi.nlm.nih.gov/books/NBK583074/. 35977056

[2] Institute of Sanimetry and Sanitary Evaluation , Global Health Data Exchange (GHDx)., 2019, http://ghdx.healthdata.org/gbd-results-tool?params=gbd-api-2019-permalink/d780dffbe8a381b25e1416884959e88b.

[3] de Sanidad M. , Subdirección General De Información Sanitaria. Salud Mental en Datos: Prevalencia De Los Problemas De Salud y Consumo De Psicofármacos y fármacos Relacionados a Partir De Registros Clínicos De Atención Primaria, 2020, https://www.sanidad.gob.es/estadEstudios/estadisticas/estadisticas/estMinisterio/SIAP/Salud_mental_datos.pdf.

[4] Cipriani A. , Furukawa T. A. , and Salanti G. , et al.Comparative Efficacy and Acceptability of 21 Antidepressant Drugs for the Acute Treatment of Adults With Major Depressive Disorder: A Systematic Review and Network Meta-Analysis, The Lancet. (2018) 391, no. 10128, 1357–1366, 10.1016/S0140-6736(17)32802-7, 2-s2.0-85043237371. PMC5889788 29477251

[5] Hu Y. , Xue H. , Ni X. , Guo Z. , Fan L. , and Du W. , Association between Duration of Antidepressant Treatment for Major Depressive Disorder and Relapse Rate after Discontinuation: A Meta-Analysis, Psychiatry Research. (2024) 337, 10.1016/j.psychres.2024.115926, 115926. 38733930

[6] Zeier Z. , Carpenter L. L. , and Kalin N. H. , et al.Clinical Implementation of Pharmacogenetic Decision Support Tools for Antidepressant Drug Prescribing, American Journal of Psychiatry. (2018) 175, no. 9, 873–886, 10.1176/appi.ajp.2018.17111282, 2-s2.0-85052067181. 29690793 PMC6774046

[7] Shekho D. , Mishra R. , Kamal R. , Khurana D. , Chauhan A. , and Awasthi A. , Polypharmacy in Psychiatry: An In-Depth Examination of Drug-Drug Interactions and Treatment Challenges, Current Pharmaceutical Design. (2024) 30, no. 21, 1641–1649, 10.2174/0113816128297170240513105418. 38798217

[8] Masnoon N. , Shakib S. , Kalisch-Ellett L. , and Caughey G. E. , What Is Polypharmacy? A Systematic Review of Definitions», BMC Geriatrics. (2017) 17, no. 1, 10.1186/s12877-017-0621-2, 2-s2.0-85031129418, 230. 29017448 PMC5635569

[9] World Health Organisation , Medication Safety in Polypharmacy, 2019, https://www.who.int/docs/default-source/patient-safety/who-uhc-sds-2019-11-eng.pdf.

[10] Kadra G. , Stewart R. , and Shetty H. , et al.Long-Term Antipsychotic Polypharmacy Prescribing in Secondary Mental Health Care and the Risk of Mortality, Acta Psychiatrica Scandinavica. (2018) 138, no. 2, 123–132, 10.1111/acps.12906, 2-s2.0-85047667206. 29845597 PMC6099447

[11] Scottish Government Model of Care Polypharmacy Working Group , Polypharmacy Guidance, 2015, 2nd Edition edition, Scottish Government, https://www.publications.scot.nhs.uk/files/dc20150415polypharmacy.pdf.

[12] Ishtiak-Ahmed K. , Köhler-Forsberg O. , Mortensen E. L. , Nierenberg A. A. , and Gasse C. , Concurrent Use of Polypharmacy and Potentially Inappropriate Medications With Antidepressants in Older Adults: A Nationwide Descriptive Study in Denmark during 2015-2019, General Hospital Psychiatry. (2023) 82, 66–74, 10.1016/j.genhosppsych.2023.03.009. 36989765

[13] Chen Y. and Ding L. , Potential Drug-Drug Interactions in Outpatients With Depression of a Psychiatry Department, Saudi Pharmaceutical Journal. (2023) 31, no. 2, 207–213, 10.1016/j.jsps.2022.12.004. 36942274 PMC10023543

[14] Kok R. M. and Reynolds C. F.III, Management of Depression in Older Adults: A Review, JAMA. (2017) 317, no. 20, 2114–2122, 10.1001/jama.2017.5706, 2-s2.0-85019603934. 28535241

[15] Davies L. E. , Spiers G. , Kingston A. , Todd A. , Adamson J. , and Hanratty B. , Adverse Outcomes of Polypharmacy in Older People: Systematic Review of Reviews, Journal of the American Medical Directors Association. (2020) 21, no. 2, 181–187, 10.1016/j.jamda.2019.10.022. 31926797

[16] Xu Q. , Ou X. , and Li J. , The Risk of Falls Among the Aging Population: A Systematic Review and Meta-Analysis, Frontiers in Public Health. (2022) 10, 10.3389/fpubh.2022.902599, 902599. 36324472 PMC9618649

[17] Infante-Ventura D. , B.D. , and n Rodríguez-Díaz , et al.Analysis of Therapeutic Adherence to Antidepressants and Associated Factors in Patients With Depressive Disorder: A Population-Based Cohort Study, Journal of Affective Disorders. (2025) 385, 10.1016/j.jad.2025.119443, 119443. 40398613

[18] De las Cuevas C. and Sanz E. J. , Polypharmacy in Psychiatric Practice in the Canary Islands, BMC Psychiatry. (2004) 4, no. 1, 10.1186/1471-244X-4-18, 2-s2.0-12944288023, 18. 15236661 PMC471555

[19] NASMHPD , 2001, *Technical Report on Psychiatric Polypharmacy*, https://www.nasmhpd.org/sites/default/files/Polypharmacy.pdf.

[20] R Core Team , R: A Language and Environment for Statistical Computing, Versión 4.3.3. R Foundation for Statistical Computing, Released2024, https://www.R-project.org/.

[21] Rhee T. G. and Rosenheck R. A. , Psychotropic Polypharmacy Reconsidered: Between-Class Polypharmacy in the Context of Multimorbidity in the Treatment of Depressive Disorders, Journal of Affective Disorders. (2019) 252, 450–457, 10.1016/j.jad.2019.04.018, 2-s2.0-85064319518. 31004825 PMC6520147

[22] Wolff J. , Reißner P. , and Hefner G. , et al.Pharmacotherapy, Drug-Drug Interactions and Potentially Inappropriate Medication in Depressive Disorders, PLoS ONE. (2021) 16, no. 7, 10.1371/journal.pone.0255192. PMC8297778 34293068

[23] Ghaed-Sharaf M. , Hariri S. , and Poustchi H. , et al.The Pattern of Medication Use, and Determinants of the Prevalence of Polypharmacy Among Patients With a Recent History of Depressive Disorder: Results From the Pars Cohort Study, BMC Psychology. (2022) 10, no. 1, 10.1186/s40359-022-00716-9, 12. 35042543 PMC8767713

[24] Delara M. , Murray L. , and Jafari B. , et al.Prevalence and Factors Associated With Polypharmacy: A Systematic Review and Meta-Analysis, BMC Geriatrics. (2022) 22, no. 1, 10.1186/s12877-022-03279-x, 601. 35854209 PMC9297624

[25] Pandi-Perumal S. R. , Monti J. M. , and Burman D. , et al.Clarifying the Role of Sleep in Depression: A Narrative Review, Psychiatry Research. (2020) 291, 10.1016/j.psychres.2020.113239, 113239. 32593854

[26] ter Meulen W. G. , Draisma S. , and van Hemert A. M. , et al.Depressive and Anxiety Disorders in Concert–A Synthesis of Findings on Comorbidity in the NESDA Study, Journal of Affective Disorders. (2021) 284, 85–97, 10.1016/j.jad.2021.02.004. 33588240

[27] Taylor R. W. , Marwood L. , and Oprea E. , et al.Pharmacological Augmentation in Unipolar Depression: A Guide to the Guidelines, International Journal of Neuropsychopharmacology. (2020) 23, no. 9, 587–625, 10.1093/ijnp/pyaa033. 32402075 PMC7710919

[28] Sarkar S. , Psychiatric Polypharmacy, Etiology and Potential Consequences, Current Psychopharmacology. (2017) 6, no. 1, 12–26, 10.2174/2211556005666160916124719.

[29] American Geriatrics Society Beers Criteria® Update Expert Panel , American Geriatrics Society 2023 Updated AGS Beers Criteria for Potentially Inappropriate Medication Use in Older Adults, Journal of the American Geriatrics Society. (2023) 71, no. 7, 2052–2081, 10.1111/jgs.18372. 37139824 PMC12478568

[30] Thunander Sundbom L. and Hedborg K. , Association Between Prescribed Antidepressants and Other Prescribed Drugs Differ by Gender: A Nationwide Register-Based Study in Sweden, Nordic Journal of Psychiatry. (2019) 73, no. 1, 73–79, 10.1080/08039488.2018.1536766, 2-s2.0-85060341279. 30661437

[31] Bacigalupe A. and Martín U. , Gender Inequalities in Depression/Anxiety and the Consumption of Psychotropic Drugs: Are we Medicalising Women’s Mental Health?, Scandinavian Journal of Public Health. (2021) 49, no. 3, 317–324, 10.1177/1403494820944736. 32755295

[32] Cebrino J. A. and de la Cruz S. P. , Environmental, Health and Sociodemographic Determinants Related to Common Mental Disorders in Adults: A Spanish Country-Wide Population-Based Study (2006-2017), Journal of Clinical Medicine. (2020) 9, no. 7, 10.3390/jcm9072199, 2199. 32664638 PMC7408656

[33] Maestre-Miquel C. , López-de-Andrés A. , and Ji Z. , et al.Gender Differences in the Prevalence of Mental Health, Psychological Distress and Psychotropic Medication Consumption in Spain: A Nationwide Population-Based Study, International Journal of Environmental Research and Public Health. (2021) 18, no. 12, 10.3390/ijerph18126350, 12. PMC8296165 34208274

[34] Milosavljevic F. , Bukvic N. , and Pavlovic Z. , et al.Association of CYP2C19 and CYP2D6 Poor and Intermediate Metabolizer Status With Antidepressant and Antipsychotic Exposure: A Systematic Review and Meta-Analysis», JAMA Psychiatry. (2021) 78, no. 3, 270–280, 10.1001/jamapsychiatry.2020.3643. 33237321 PMC7702196

[35] Bousman C. A. , Stevenson J. M. , and Ramsey L. B. , et al.Clinical Pharmacogenetics Implementation Consortium (CPIC) Guideline for *CYP2D6*, *CYP2C19*, *CYP2B6*, *SLC6A4*, and *HTR2A* Genotypes and Serotonin Reuptake Inhibitor Antidepressants, Clinical Pharmacology & Therapeutics. (2023) 114, no. 1, 51–68, 10.1002/cpt.2903. 37032427 PMC10564324

[36] Fabbri C. , Tansey K. E. , and Perlis R. H. , et al.Effect of Cytochrome CYP2C19 Metabolizing Activity on Antidepressant Response and Side Effects: Meta-Analysis of Data From Genome-Wide Association Studies, European Neuropsychopharmacology. (2018) 28, no. 8, 945–954, 10.1016/j.euroneuro.2018.05.009, 2-s2.0-85049099531. 30135031

[37] Thiele L. S. , Ishtiak-Ahmed K. , Thirstrup J. P. , Agerbo E. , Lunenburg C. A. T. C. , Müller D. J. , and Gasse C. , Clinical Impact of Functional CYP2C19 and CYP2D6 Gene Variants on Treatment With Antidepressants in Young People With Depression: A Danish Cohort Study, Pharmaceuticals. (2022) 15, no. 7, 10.3390/ph15070870, 870. 35890168 PMC9318115

[38] Marshe V. S. , Islam F. , and Maciukiewicz M. , et al.Pharmacogenetic Implications for Antidepressant Pharmacotherapy in Late-Life Depression: A Systematic Review of the Literature for Response, Pharmacokinetics and Adverse Drug Reactions, The American Journal of Geriatric Psychiatry. (2020) 28, no. 6, 609–629, 10.1016/j.jagp.2020.01.007. 32122803

[39] LeGates T. A. , Kvarta M. D. , and Thompson S. M. , Sex Differences in Antidepressant Efficacy, Neuropsychopharmacology. (2019) 44, no. 1, 140–154, 10.1038/s41386-018-0156-z, 2-s2.0-85052913611. 30082889 PMC6235879

[40] Fernández-Guasti A. , Fiedler J. , Herrera L. , and Handa R. , Sex, Stress, and Mood Disorders: At the Intersection of Adrenal and Gonadal Hormones, Hormone and Metabolic Research. (2012) 44, no. 8, 607–618, 10.1055/s-0032-1312592, 2-s2.0-84863722805. 22581646 PMC3584173

[41] Bigos K. L. , Pollock B. G. , Stankevich B. A. , and Bies R. R. , Sex Differences in the Pharmacokinetics and Pharmacodynamics of Antidepressants: An Updated Review, Gender Medicine. (2009) 6, no. 4, 522–543, 10.1016/j.genm.2009.12.004, 2-s2.0-74549176824. 20114004

[42] Dalla C. , Pitychoutis P. M. , Kokras N. , and Papadopoulou-Daifoti Z. , Sex Differences in Animal Models of Depression and Antidepressant Response, Basic & Clinical Pharmacology & Toxicology. (2010) 106, no. 3, 226–233, 10.1111/j.1742-7843.2009.00516.x, 2-s2.0-77149123942. 20050844

[43] Correll C. U. , Frederickson A. M. , Kane J. M. , and Manu P. , Does Antipsychotic Polypharmacy Increase the Risk for Metabolic Syndrome?, Schizophrenia Research. (2007) 89, no. 1–3, 91–100, 10.1016/j.schres.2006.08.017, 2-s2.0-33845320133. 17070017 PMC2718048

[44] Gordon P. , Louza M. R. , and Xavier , Weight Gain, Metabolic Disturbances, and Physical Health Care in a Brazilian Sample of Outpatients With Schizophrenia, Neuropsychiatric Disease and Treatment. (2013) 9, 133–138, 10.2147/NDT.S37019, 2-s2.0-84872859043. 23355783 PMC3552546

[45] Mizokami F. , Koide Y. , Noro T. , and Furuta K. , Polypharmacy With Common Diseases in Hospitalized Elderly Patients, The American Journal of Geriatric Pharmacotherapy. (2012) 10, no. 2, 123–128, 10.1016/j.amjopharm.2012.02.003, 2-s2.0-84859529323. 22387105

[46] Larco J. P. and Jeste D. V. , Physical Comorbidity and Polypharmacy in Older Psychiatric Patients, Biological Psychiatry. (1994) 36, no. 3, 146–152, 10.1016/0006-3223(94)91220-3, 2-s2.0-0028111908. 7948452

[47] Kroken R. A. , Johnsen E. , Ruud T. , Wentzel-Larsen T. , and Jørgensen H. A. , Treatment of Schizophrenia with Antipsychotics in Norwegian Emergency Wards, a Cross-Sectional National Study, BMC Psychiatry. (2009) 9, no. 1, 10.1186/1471-244X-9-24, 2-s2.0-67649695935, 24. 19445700 PMC2693495

[48] Clark R. E. , Xie H. , and Brunette M. F. , Benzodiazepine Prescription Practices and Substance Abuse in Persons With Severe Mental Illness, The Journal of Clinical Psychiatry. (2004) 65, no. 2, 151–155, 10.4088/JCP.v65n0202, 2-s2.0-1842844964. 15003066

[49] Brunette M. F. , Noordsy D. L. , Xie H. , and Drake R. E. , Benzodiazepine Use and Abuse Among Patients With Severe Mental Illness and Co-Occurring Substance Use Disorders, Psychiatric Services. (2003) 54, no. 10, 1395–1401, 10.1176/appi.ps.54.10.1395, 2-s2.0-0141842763. 14557527

[50] Hemmert G. A. J. , Schons L. M. , Wieseke J. , and Schimmelpfennig H. , Log-Likelihood-Based Pseudo-R^2^ in Logistic Regression: Deriving Sample-Sensitive Benchmarks, Sociological Methods & Research. (2018) 47, no. 3, 507–531, 10.1177/0049124116638107, 2-s2.0-85050007004.

[51] Smith T. J. and McKenna C. M. , A Comparison of Logistic Regression Pseudo R2 Indices, Multiple Linear Regression Viewpoints. (2013) 39, no. 2, 17–26.

[52] Mann N.-K. , Mathes T. , Sönnichsen A. , Pieper D. , Klager E. , Moussa M. , and Thürmann P. A. , Potentially Inadequate Medications in the Elderly: PRISCUS. 2.0, Deutsches Ärzteblatt International. (2023) 120, no. 1-2, 3–10, 10.3238/arztebl.m2022.0377. 36507719 PMC10035347

[53] Munkholm K. , Ussing A. , and Brink M. , et al.Minor Tranquillizers for Short-Term Treatment of Newly Onset Symptoms of Anxiety and Distress: A Systematic Review With Network Meta-Analysis of Randomized Trials, European Archives of Psychiatry and Clinical Neuroscience. (2024) 274, no. 3, 475–486, 10.1007/s00406-023-01680-0. 37624378 PMC10995039

[54] Davies J. , Rae T. C. , and Montagu L. , Long-Term Benzodiazepine and Z-Drugs Use in England: A Survey of General Practice, British Journal of General Practice. (2017) 67, no. 662, e609–e613, 10.3399/bjgp17X691865, 2-s2.0-85031284007. PMC5569740 28716996

[55] Tähkäpää S.-M. , Saastamoinen L. , Airaksinen M. , Tuulio-Henriksson A. , Aalto-Setälä T. , and Kurko T. , Decreasing Trend in the Use and Long-Term Use of Benzodiazepines Among Young Adults, Journal of Child and Adolescent Psychopharmacology. (2018) 28, no. 4, 279–284, 10.1089/cap.2017.0140, 2-s2.0-85046938291. 29641240

[56] Martella M. , Minutiello E. , and Gianino M. M. , Consumption and Expenditure of Antidepressants and Anxiolitycs in 14 European Countries, European Journal of Public Health. (2024) 34, no. Supplement_3, 10.1093/eurpub/ckae144.185, 185.

[57] Sackett D. L. , Rosenberg W. M. C. , Gray J. A. M. , Haynes R. B. , and Richardson W. S. , Evidence Based Medicine: What It Is and What it Isn’t”, BMJ. (1996) 312, no. 7023, 71–72, 10.1136/bmj.312.7023.71. 8555924 PMC2349778

