# Supplementary material for: Analysis of Psychotropic Polypharmacy and Associated Factors in Antidepressant‐Treated Patients With Depressive Disorder: A Population‐Based Cohort Study Using Real World Data
Source: Depress Anxiety. 2026 Apr 27;2026:6076326. doi: 10.1155/da/6076326 (PMC13121550; doi:10.1155/da/6076326)
Supplement: Supplementary file 1 — Supporting Information The supporting information includes the STROBE Statement (Table S1) and correspondence tables between ICD‐10 and ICD‐9 diagnostic codes for depressive disorders (Table S2), manic episode and bipolar disorder (Table S3), and schizophrenia, schizotypal disorder, and delusional disorders (Table S4). Furthermore, Supporting Table S5 provides a distribution of antidepressant use by prescription and by patient, with an analysis according to psychotropic polypharmacy/no‐polypharmacy. [file DA-2026-6076326-s001.docx]

**Supplementary Table S1. STROBE Statement—Checklist of items that should be included in reports of *cohort studies*****.**

|  | Item No | Recommendation | Comment |
| --- | --- | --- | --- |
| Title and abstract | 1 | (*a*) Indicate the study’s design with a commonly used term in the title or the abstract | Title: *“a population-based cohort study”*; Abstract: *“This is a population-based retrospective cohort study”*. |
|  |  | (*b*) Provide in the abstract an informative and balanced summary of what was done and what was found | See Abstract |
| Introduction | | |  |
| Background/rationale | 2 | Explain the scientific background and rationale for the investigation being reported | See the “*1. Introduction*” section |
| Objectives | 3 | State specific objectives, including any prespecified hypotheses | See last paragraph of the “*1. Introduction*” section |
| Methods | | |  |
| Study design | 4 | Present key elements of study design early in the paper | See the *“2.1. Design and setting”* subsection |
| Setting | 5 | Describe the setting, locations, and relevant dates, including periods of recruitment, exposure, follow-up, and data collection | See the *“2.1. Design and setting”* subsection |
| Participants | 6 | (*a*) Give the eligibility criteria, and the sources and methods of selection of participants. Describe methods of follow-up | See the *“2.2. Participants”* subsection |
|  |  | (*b*) For matched studies, give matching criteria and number of exposed and unexposed | Not applicable |
| Variables | 7 | Clearly define all outcomes, exposures, predictors, potential confounders, and effect modifiers. Give diagnostic criteria, if applicable | See the *“2.3. Variables”* subsection |
| Data sources/ measurement | 8 | For each variable of interest, give sources of data and details of methods of assessment (measurement). Describe comparability of assessment methods if there is more than one group | See the *“2.4. Variables”* subsection |
| Bias | 9 | Describe any efforts to address potential sources of bias | See the *"4.1. Strengths and limitations of the study"* subsection |
| Study size | 10 | Explain how the study size was arrived at | It is based on a population-based cohort design |
| Quantitative variables | 11 | Explain how quantitative variables were handled in the analyses. If applicable, describe which groupings were chosen and why | See the *“2.4. Variables”* and *“2.5. Analysis”* subsections |
| Statistical methods | 12 | (*a*) Describe all statistical methods, including those used to control for confounding | See the *“2.5. Analysis”* subsections |
|  |  | (*b*) Describe any methods used to examine subgroups and interactions | See the *“2.5. Analysis”* subsections |
|  |  | (*c*) Explain how missing data were addressed | See the *“2.5. Analysis”* subsections |
|  |  | (*d*) If applicable, explain how loss to follow-up was addressed | Not applicable |
|  |  | (*e*) Describe any sensitivity analyses | Not applicable |
| Results | | |  |
| Participants | 13 | (a) Report numbers of individuals at each stage of study—eg numbers potentially eligible, examined for eligibility, confirmed eligible, included in the study, completing follow-up, and analysed | See first paragraph of the “*3. Results*” section, *“A total of 39,800 participants […] analyzed 693,686 prescriptions in total”.* |
|  |  | (b) Give reasons for non-participation at each stage | Not applicable |
|  |  | (c) Consider use of a flow diagram | See in *“Fig. 1. Flowchart of selection process”* |
| Descriptive data | 14 | (a) Give characteristics of study participants (eg demographic, clinical, social) and information on exposures and potential confounders | See Tables 1, 2, and 3 of the manuscript for characteristics of the study participants. |
|  |  | (b) Indicate number of participants with missing data for each variable of interest | See “*Table 1. Characteristics of participants and prevalence of psychiatric polypharmacy”.* |
|  |  | (c) Summarise follow-up time (eg, average and total amount) | See “*Table 1. Characteristics of participants and prevalence of psychiatric polypharmacy”.* |
| Outcome data | 15 | Report numbers of outcome events or summary measures over time | See the *“3. Results”* section |
| Main results | 16 | (*a*) Give unadjusted estimates and, if applicable, confounder-adjusted estimates and their precision (eg, 95% confidence interval). Make clear which confounders were adjusted for and why they were included | An adjusted estimate was provided in *"Table 5. Factors associated with polypharmacy"* and a description of the factors that were adjusted and why they were included was provided in the *"2.5. Analysis" subsection.* |
|  |  | (*b*) Report category boundaries when continuous variables were categorized | See the *"Characteristics"* column of *"Table 5. Factors associated with polypharmacy"* |
|  |  | (*c*) If relevant, consider translating estimates of relative risk into absolute risk for a meaningful time period | Not applicable |
| Other analyses | 17 | Report other analyses done—eg analyses of subgroups and interactions, and sensitivity analyses | *See Table 3 and Table 4.* Subgroup analyses were performed by status (polypharmacy and non-polypharmacy) and by type of polypharmacy (with-on, between and both). |
| Discussion | | |  |
| Key results | 18 | Summarise key results with reference to study objectives | See the *"4. Discussion"* section |
| Limitations | 19 | Discuss limitations of the study, taking into account sources of potential bias or imprecision. Discuss both direction and magnitude of any potential bias | See the *"4.1. Strengths and limitations of the study"* subsection |
| Interpretation | 20 | Give a cautious overall interpretation of results considering objectives, limitations, multiplicity of analyses, results from similar studies, and other relevant evidence | See the *"4. Discussion" section* |
| Generalisability | 21 | Discuss the generalisability (external validity) of the study results | See last paragraph of the *"4. Discussion"* section |
| Other information | | |  |
| Funding | 22 | Give the source of funding and the role of the funders for the present study and, if applicable, for the original study on which the present article is based | See the *“5.3. Funding”* subsection |

**Supplementary Table S2. ICD-10 to ICD-9 Mapping: Depressive Disorders (ICD-10: F32-F33, Depressive episodes and Recurrent depressive disorder; F34.1, Dysthymia).**

| **ICD-9 codes** | **Depressive episodes and Recurrent depressive disorder** |
| --- | --- |
| 296.2 | Major depressive disorder single episode |
| 296.20 | Major depressive disorder, single episode, unspecified |
| 296.21 | Major depressive disorder, single episode, mild |
| 296.22 | Major depressive disorder, single episode, moderate |
| 296.23 | Major depressive disorder, single episode, severe, without mention of psychotic behavior |
| 296.24 | Major depressive disorder, single episode, severe, specified as with psychotic behavior |
| 296.25 | Major depressive disorder, single episode, in partial or unspecified remission |
| 296.26 | Major depressive disorder, single episode, in full remission |
| 296.3 | Major depressive disorder recurrent episode |
| 296.30 | Major depressive disorder, recurrent episode, unspecified |
| 296.31 | Major depressive disorder, recurrent episode, mild |
| 296.32 | Major depressive disorder, recurrent episode, moderate |
| 296.33 | Major depressive disorder, recurrent episode, severe, without mention of psychotic behavior |
| 296.34 | Major depressive disorder, recurrent episode, severe, specified as with psychotic behavior |
| 296.35 | Major depressive disorder, recurrent episode, in partial or unspecified remission |
| 296.36 | Major depressive disorder, recurrent episode, in full remission |
| 296.82 | Atypical depressive disorder |
| 296.9 | Other and unspecified episodic mood disorder |
| 296.90 | Unspecified episodic mood disorder |
| 296.99 | Other specified episodic mood disorders |
| 301.10 | Affective personality disorder, unspecified |
| 301.12 | Chronic depressive personality disorder |
| 301.13 | Cyclothymic disorder |
| 300.4 | Dysthymic disorder |
| 311 | Depressive disorder, not elsewhere classified |
| **ICD-9: Ninth Revision of International Classification of Diseases** | |

**Supplementary Table S3. ICD-10 to ICD-9 Mapping: Maniac episode and Bipolar disorder (ICD-10: F30-F31).**

| **ICD-9 codes** | **Maniac episode and Bipolar disorder** | |
| --- | --- | --- |
| 296.00 | Bipolar I disorder, single manic episode, unespecified | |
| 296.01 | Bipolar I disorder, single manic episode, mild | |
| 296.02 | Bipolar I disorder, single manic episode, moderate | |
| 296.03 | Bipolar I disorder, single manic episode grave, without mention of psychotic behavior | |
| 296.04 | Bipolar I disorder, single manic episode grave, specified as with psychotic behavior | |
| 296.05 | Bipolar I disorder, single manic, in partial or unspecified remission | |
| 296.06 | Bipolar I disorder, single manic episode, in full remission | |
| 296.40 | Bipolar I disorder, most recent episode (or current), maniac, unspecified | |
| 296.41 | Bipolar I disorder, most recent episode (or current), maniac, mild | |
| 296.42 | Bipolar I disorder, most recent episode (or current), maniac, moderate | |
| 296.43 | Bipolar I disorder, most recent episode (or current), maniac, severe, without mention of psychotic behavior | |
| 296.44 | Bipolar I disorder, most recent episode (or current), maniac, severe, specified as with psychotic behavior | |
| 296.45 | Bipolar I disorder, most recent episode (or current), maniac, in partial or unspecified remission | |
| 296.46 | Bipolar I disorder, most recent episode (or current), maniac, in full remission | |
| 296.50 | Bipolar I disorder, most recent episode (or current) depressed, unspecified | |
| 296.51 | Bipolar I disorder, most recent episode (or current) depressed, mild | |
| 296.52 | Bipolar I disorder, most recent episode (or current) depressed, moderate | |
| 296.53 | Bipolar I disorder, most recent episode (or current), severe, without mention of psychotic behavior | |
| 296.54 | Bipolar I disorder, most recent episode (or current), severe, specified as with psychotic behavior | |
| 296.55 | Bipolar I disorder, most recent episode (or current), in partial or unspecified remission | |
| 296.56 | Bipolar I disorder, most recent episode (or current), in full remission | |
| 296.60 | Bipolar I disorder, most recent episode (or current) mixed, unspecified | |
| 296.61 | Bipolar I disorder, most recent episode (or current) mixed, mild | |
| 296.62 | Bipolar I disorder, most recent episode (or current) mixed, moderate | |
| 296.63 | Bipolar I disorder, most recent episode (or current) mixed, severe, without mention of psychotic behavior | |
| 296.64 | Bipolar I disorder, most recent episode (or current) mixed, severe, specified as with psychotic behavior | |
| 296.65 | Bipolar I disorder, most recent episode (or current) mixed, in partial or unspecified remission | |
| 296.66 | Bipolar I disorder, most recent episode (or current) mixed, in full remission | |
| 296.7 | Bipolar I disorder, most recent episode (or current) unspecified | |
| 296.80 | Bipolar disorder, unspecified | |
| 296.81 | Atypical manic disorder | |
| 296.89 | Other bipolar disorders | |
| ICD-9: Ninth Revision of International Classification of Diseases | |  |

**Supplementary Table S4. ICD-10 to ICD-9 Mapping: Schizophrenia, Schizotypal disorder, or Delusional disorders (ICD-10: F20-F29).**

| **ICD-9 Code** | **Schizophrenia, Schizotypal disorder, or Delusional disorders** |
| --- | --- |
| 295.10 | Disorganized type schizophrenia, unspecified |
| 295.20 | Catatonic type schizophrenia, unspecified |
| 295.30 | Paranoid type schizophrenia, unspecified |
| 295.40 | Acute schizophrenic episode, unspecified |
| 295.60 | Residual schizophrenia, unspecified |
| 295.70 | Schizo-affective type schizophrenia, unspecified |
| 295.80 | Other specified types of schizophrenia, unspecified |
| 295.90 | Unspecified schizophrenia |
| 297.0 | Paranoid state, simple |
| 297.1 | Delusional disorder |
| 297.2 | Paraphrenia |
| 297.3 | Shared psychotic disorder |
| 298.3 | Acute paranoid reaction |
| 298.4 | Psychogenic paranoid psychosis |
| 298.8 | Other and unspecified reactive psychosis |
| 298.9 | Unspecified psychosis |
| 301.22 | Schizotypal personality disorder |
| ICD-9: Ninth Revision of International Classification of Diseases | |

**Supplementary Table S5. Distribution of antidepressant use per prescriptions and per patients (by psychiatric polypharmacy).**

| **Group description (ATC code)** | **Per prescriptions**  **N = 366 415 (%; 95% CI)** | **Per patients**  **N = 39 800 (%; 95% CI)** | **Polypharmacy**  **N = 34 338** **(%; 95% CI)** | **No polypharmacy**  **N = 5462** **(%; 95% CI)** |
| --- | --- | --- | --- | --- |
| Non-Selective Monoamine Reuptake Inhibitors (N06AA) | 19 140 (5.22; 5.15 - 5.30) | 3467 (8.71; 8.44 - 8.99) | 3111 (9.06; 8.76 - 9.37) | 356 (6.52; 5.88 - 7.21) |
| Imipramine (N06AA02) | 149 (0.041; 0.035 - 0.048) | 38 (0.095; 0.069 - 0.132) | 34 (0.10; 0.07 - 0.14) | 4 (0.07; 0.02 - 0.20) |
| Clomipramine (N06AA04) | 2849 (0.778; 0.749 - 0.807) | 541 (1.36; 1.25 - 1.48) | 526 (1.53; 1.41 - 1.67) | 15 (0.27; 0.16 - 0.46) |
| Trimipramine (N06AA06) | 22 (0.006; 0.004 - 0.009) | 5 (0.013; 0.005 - 0.031) | 5 (0.01; 0.01- 0.04) | 0 |
| Amitriptyline (N06AA09) | 15 315 (4.18; 4.12 - 4.25) | 2825 (7.10; 6.85 - 7.36) | 2497 (7.27; 7.00 - 7.56) | 328 (6.01; 5.40 - 6.68) |
| Nortriptyline (N06AA10) | 295 (0.081; 0.072 - 0.090) | 50 (0.126; 0.094 - 0.167) | 46 (0.13; 0.09 - 0.18) | 4 (0.07; 0.02 - 0.20) |
| Doxepin (N06AA12) | 180 (0.049; 0.042 - 0.057) | 34 (0.085; 0.060 - 0.121) | 29 (0.08; 0.06 - 0.12) | 5 (0.09; 0.03 - 0.23) |
| Maprotiline (N06AA21) | 330 (0.09; 0.08 - 0.10) | 75 (0.188; 0.149 - 0.238) | 73 (0.21; 0.17 - 0.27) | 2 (0.04; 0.01 - 0.15) |
| SSRIs (N06AB) | 164 191 (44.81; 44.65 - 44.97) | 27 573 (69.28; 68.82 - 69.73) | 23 979 (69.83; 69.34 - 70.33) | 3594 (65.80; 64.52 - 67.06) |
| Fluoxetine (N06AB03) | 21 897 (5.98; 5.90 - 6.05) | 4364 (10.97; 10.66 - 11.28) | 3798 (11.06; 10.73 - 11.40) | 566 (10.36; 9.57 - 11.21) |
| Citalopram (N06AB04) | 20 865 (5.69; 5.62 - 5.77) | 4214 (10.59; 10.29 - 10.90) | 3634 (10.58; 10.26 - 10.91) | 580 (10.62; 9.82 - 11.47) |
| Paroxetine (N06AB05) | 32 178 (8.78; 8.69 - 8.87) | 6222 (15.63; 15.28 - 16.00) | 5572 (16.23; 15.84 - 16.62) | 650 (11.90; 11.06 - 12.80) |
| Sertraline (N06AB06) | 50 481 (13.78; 13.67 - 13.89) | 8878 (22.31; 21.90 - 22.72) | 7706 (22.44; 22.00 - 22.89) | 1172 (21.46; 20.38 - 22.58) |
| Fluvoxamine (N06AB08) | 781 (0.213; 0.199 - 0.229) | 138 (0.35; 0.29 - 0.41) | 129 (0.38; 0.32 - 0.45) | 9 (0.16; 0.08 - 0.33) |
| Escitalopram (N06AB10) | 37 989 (10.37; 10.27 - 10.47) | 7255 (18.23; 17.85 - 18.61) | 6462 (18.82; 18.41- 19.24) | 793 (14.52; 13.60 - 15.49) |
| Other Antidepressants (N06AX) | 183 084 (49.97; 49.80 - 50.13) | 21 930 (55.10; 54.61 - 55.59) | 20 117 (58.59; 58.06 - 59.11) | 1813 (33.19; 31.95 - 34.46) |
| Mianserin (N06AX03) | 2657 (0.725; 0.698 - 0.753) | 479 (1.20; 1.10 - 1.32) | 453 (1.32; 1.20 - 1.45) | 26 (0.48; 0.32 - 0.71) |
| Trazodone (N06AX05) | 41 292 (11.27; 11.17 - 11.37) | 6954 (17.47; 17.10 - 17.85) | 6632 (19.31; 18.90 - 19.74) | 322 (5.90; 5.29 - 6.56) |
| Mirtazapine (N06AX11) | 35 540 (9.70; 9.60 - 9.80) | 6104 (15.34; 14.99 - 15.70) | 5795 (16.88; 16.48 - 17.28) | 309 (5.66; 5.07 - 6.31) |
| Bupropion (N06AX12) | 5370 (1.47; 1.43 - 1.51) | 1116 (2.80; 2.65 - 2.97) | 1045 (3.04; 2.87 - 3.23) | 71 (1.30; 1.02 - 1.16) |
| Tianeptine (N06AX14) | 756 (0.206; 0.192 - 0.222) | 148 (0.37; 0.32 - 0.44) | 144 (0.42; 0.36 - 0.50) | 4 (0.07; 0.02 - 0.20) |
| Venlafaxine (N06AX16) | 25 713 (7.02; 6.94 - 7.10) | 4272 (10.73; 10.43 - 11.04) | 4020 (11.71; 11.37 - 12.05) | 252 (4.61; 4.08 - 5.21) |
| Reboxetine (N06AX18) | 731 (0.200; 0.185 - 0.215) | 148 (0.37; 0.32 - 0.44) | 144 (0.42; 0.36 - 0.50) | 4 (0.07; 0.02 - 0.20) |
| Duloxetine (N06AX21) | 30 910 (8.44; 8.35 - 8.53) | 5107 (12.83; 12.51 - 13.17) | 4698 (13.68; 13.32 - 14.05) | 409 (7.49; 6.81 - 8.23) |
| Agomelatine (N06AX22) | 7186 (1.96; 1.92 - 2.01) | 1475 (3.71; 3.52 - 3.90) | 1400 (4.08; 3.87 - 4.29) | 75 (1.37; 1.09 - 1.73) |
| Desvenlafaxine (N06AX23) | 23 141 (6.32; 6.24 - 6.40) | 4329 (10.88; 10.57 - 11.19) | 4051 (11.80; 11.46 - 12.14) | 278 (5.09; 4.53 - 5.71) |
| Vortioxetine (N06AX26) | 9788 (2.67; 2.62 - 2.72) | 1696 (4.26; 4.07 - 4.47) | 1553 (4.52; 4.31 - 4.75) | 143 (2.62; 2.22 - 3.09) |
| *CI 95%: 95% confidence interval; N: number of patients; SSRIs: Selective Serotonin Reuptake Inhibitors.*  *Note: the last three columns represent the total number of patients who consumed that medication at any point during the year* | | | | |

**Supplementary Table S6. List of prescribed or dispensed medicines**

N03A - Antiepileptic drugs

N03AF01 – Carbamazepine

N03AG01 – Valproic acid

N03AX09 – Lamotrigine

N05A – Antipsychotics

N05AA – Phenothiazines with aliphatic side chain

N05AA01 – Chlorpromazine

N05AA02 - Levomepromazine

N05AB – Phenothiazines with piperazine structure

N05AB02 – Fluphenazine

N05AB03 - Perphenazine

N05AB06 – Trifluoperazine

N05AB08 – Thioproperazine

N05AC – Phenothiazines with piperidine structure

N05AC01 - Periciazine

N05AC02 - Thioridazine

N05AC04 – Pipotiazine

N05AD – Butyrophenone derivatives

N05AD01 - Haloperidol

N05AD06 - Bromperidol

N05AD08 – Droperidol

N05AE – Indole derivatives

N05AE03 - Sertindole

N05AE04 - Ziprasidone

N05AE05 – Lurasidone

N05AF – Thioxanthene derivatives

N05AF01 - Flupentixol

N05AF03 - Chlorprothixene

N05AF05 - Zuclopenthixol

N05AG – Diphenylbutylpiperidine derivatives

N05AG02 - Pimozida

N05AG03 – Penfluridol

N05AH – Diazepines, oxazepines, thiazepines, and oxepines

N05AH1 – Loxapine

N05AH2 - Clozapine

N05AH3 - Olanzapine

N05AH4 - Quetiapine

N05AH5 - Asenapine

N05AL – Benzamides

N05AL P1 – Sulpiride + pyridoxine

N05AL01 – Sulpiride

N05AL03 – Tiapride

N05AL05 - Amisulpride

N05AL06 - Veralipride

N05AL07 – Levosulpiride

N05AN – Lithium

N05AX – Other antipsychotics

N05AX08 - Risperidone

N05AX12 – Aripiprazole

N05AX13 - Paliperidone

N05AX14 - Iloperidone

N05AX15 - Cariprazine

N05AX16 - Brexpiprazole

N05B – Anxiolytics

N05BA – Benzodiazepine derivatives

N05BA P1 – Sulpiride + diazepam

N05BA P2 – Diazepam + pyridoxine

N05BA P3 – Potassium clorazepate + gamma amino butyric acid + pyridoxine

N05BA P4 – Sulpiride + diazepam + pyridoxine

N05BA P5 – Alprazolam + sulpiride

N05BA P6 – Alprazolam + isopropamide

N05BA01 – Diazepam

N05BA02 – Chlordiazepoxide

N05BA04 – Oxazepam

N05BA05 – Potassium clorazepate

N05BA06 – Lorazepam

N05BA08 – Bromazepam

N05BA09 – Clobazam

N05BA10 – Ketazolam

N05BA12 – Alprazolam

N05BA13 – Halazepam

N05BA14 – Pinazepam

N05BA18 – Ethyl loflazepate

N05BA21 – Clotiazepam

N05BA24 – Bentazepam

N05BB – Diphenylmethane derivatives

N05BB01 – Hydroxyzine

N05BC – Carbamates

N05BC51 – Meprobamate combinations

N05BC51 P1 - Piroxicam + prednisone + meprobamate

N05BC51 P2 - Meprobamate + piroxicam

N05BE – Azaspirodecanedione derivatives

N05BE01 – Buspirone

N05BX – Other anxiolytics

N05BX03 – Etifoxine

N05BX05 – Lavandulae aetheroleum

N05C – Hypnotics and sedatives

N05CA – Barbiturates, single

N05CA01 – Pentobarbital

N05CB01 – Barbiturates, combinations

N05CB01 P1 – Phenobarbital + pentobarbital

N05CB02 – Barbiturates in combination with other drugs

N05CB02 P1 – Phenobarbital + adifenine

N05CD – Benzodiazepine derivatives

N05CD01 – Flurazepam

N05CD03 – Flunitrazepam

N05CD05 - Triazolam

N05CD06 - Lormetazepam

N05CD08 - Midazolam

N05CD09 - Brotizolam

N05CD10 - Quazepam

N05CD11 – Loprazolam

N05CF – Drugs related to benzodiazepines

N05CF01 - Zopiclone

N05CF02 - Zolpidem

N05CF03 - Zaleplon

N05CF04 – Eszopiclone

N05CH – Melatonin receptor agonists

N05CH01 – Melatonin

N05CM – Other hypnotics and sedatives

N05CM M2 – Diphenhydramine hydrochloride

N05CM M3 – Magnesium glutamate bromhydrate

N05CM M4 – Passiflora incarnata dry extract

N05CM M5 – Hawthorn dry extract

N05CM M6 – Eschscholzia californica

N05CM M7 - Humulus lupulus

N05CM M8 – Melissa officinalis

N05CM M02 - Clomethiazole

N05CM M09 – Valerian root

N05CM M018 - Dexmedetomidine

N05CX – Hypnotics and sedatives in combination, excluding barbiturates

N05CX P1 – Valerian dry extract + melissa dry extract

N05CX P2 – Valerian dry extract + passiflora incarnata dry extract

N05CX P3 – Valerian dry extract + hops dry extract

N06A – Antidepressants

N06AA – Non-selective monoamine reuptake inhibitors

N06AA01 - Desipramine

N06AA02 - Imipramine

N06AA04 - Clomipramine

N06AA06 - Trimipramine

N06AA09 - Amitriptyline

N06AA10 - Nortriptyline

N06AA12 - Doxepin

N06AA16 - Dosulepin

N06AA21 – Maprotiline

N06AB – Selective serotonin reuptake inhibitors

N06AB P1 - Paroxetine + alprazolam

N06AB P2 - Sertraline + alprazolam

N06AB03 - Fluoxetine

N06AB04 - Citalopram

N06AB05 - Paroxetine

N06AB06 - Sertraline

N06AB08 - Fluvoxamine

N06AB10 – Escitalopram

N06AF – Non-selective monoamine oxidase inhibitors

N06AF04 – Tranylcypromine

N06AG – Monoamine oxidase A inhibitors

N06AG02 – Moclobemide

N06AX – Other antidepressants

N06AX01 - Oxitriptan

N06AX03 - Mianserin

N06AX05 - Trazodone

N06AX11 - Mirtazapine

N06AX12 - Bupropion

N06AX14 - Tianeptine

N06AX16 - Venlafaxine

N06AX17 - Milnacipran

N06AX18 - Reboxetine

N06AX21 - Duloxetine

N06AX22 - Agomelatine

N06AX23 - Desvenlafaxine

N06AX25 - St. John's Wort (Hyperici herba)

N06AX26 - Vortioxetine

N06C – Psychoanaleptics and psycholeptics in combination

N06CA – Antidepressants in combination with psycholeptics

N06CA P2 - Nortriptyline + diazepam

N06CA P3 - Nortriptyline + perphenazine

N06CA01 - Amitriptyline and psycholeptics

N06CA02 - Melitracen and psycholeptics

N06CA03 - Fluoxetine and psycholeptics

N06CB – Psychostimulants in combination with psycholeptics

N06CB P1 - Chlorpromazine + trihexyphenidyl + heptaminol
